# Evaluation of the reproductive health curriculum at medical schools in Germany: an insight into medical students’ knowledge and opinion towards emergency contraception and abortion - a cross-sectional study

**DOI:** 10.1186/s12889-025-24492-4

**Published:** 2025-09-24

**Authors:** Cecilia Rees, Andrea Kaifie

**Affiliations:** 1 Institute for Occupational, Social, and Environmental Medicine, RWTH Aachen, Aachen, Germany; 2 Institute and Outpatient Unit for Occupational, Social, and Environmental Medicine, FAU Erlangen-Nürnberg, Erlangen, Germany

**Keywords:** Survey, Reproductive health, Teaching, Gynaecology, Abortion, Emergency contraception

## Abstract

**Objectives:**

The legal framework in Germany allows patients to access abortion services only under certain regulations without being legally prosecuted. There is a decline in abortion providers, despite abortion numbers remaining the same. This trend has been associated with the legal framework as well as the limited opportunity to learn about reproductive health topics during medical school. This study therefore aimed to evaluate the medical school curriculum regarding emergency contraception and abortion by conducting a study targeted at medical students in Germany.

**Study design:**

A cross-sectional study was conducted to assess German medical students’ opinion, knowledge and curriculum evaluation towards emergency contraception and abortion using a 70-item online survey.

**Results:**

The responses of *n* = 1167 medical students from 34 German medical schools were analysed. The results showed that 97.3% of participants supported the statement that abortion should be legal. 76.8% of respondents did not believe that access to abortion in Germany is sufficient. Curriculum evaluations showed that many students were not satisfied with the curriculum offered at their faculty or had not been taught about abortion or emergency contraception. 82.4% of respondents indicated their willingness to offer abortion care in their future career.

**Conclusion:**

German medical students hold a primarily positive attitude towards abortion and show willingness to offer abortion care. However, the evaluation of the curriculum offered within their studies indicates a need for improvement, to sufficiently represent abortion and emergency contraception, with the aim of training and educating future abortion providers in accordance with current medical guidelines.

**Supplementary information:**

The online version contains supplementary material available at 10.1186/s12889-025-24492-4.

## Introduction

In Germany, just over 100.000 abortions were performed in 2022 [1]. Under current German criminal law, abortion is primarily defined as unlawful (§ 218), but is exempt from criminal prosecution in specific circumstances within a ‘conflict situation’(§ 218a): People seeking an abortion must undergo mandatory counselling (‘conflict counselling’) and a mandatory waiting period of three working days; No more than 12 weeks can have passed since conception [2, 3]. The procedure is not covered by medical insurance. 96% of abortions fall under these circumstances [1]. Terminations of pregnancy out of medical necessity or resulting from criminal acts are exempt under § 218a and are thus not categorised as unlawful. Further clarifications to these regulations can be found in the ‘Pregnancy Conflict Act’, a federal law that regulates how pregnancies in ‘conflict situations’ are to be handled [4]. On the 24th of June 2022, article § 219a, a law prohibiting doctors from publicly providing information on abortion methods or indicating whether they provided the procedure, was abolished [5].

Article § 218 specifies, that acts taking effect before the implantation of a fertilised egg in the uterus are not considered an abortion, referring to emergency contraception (EC) methods. EC only became available without a doctor’s prescription in 2015, making Germany one of the last European countries to make EC available over the counter [6]. As a result, the responsibility for EC care and counselling is shared amongst primary care physicians and pharmacists [7, 8]. According to WHO guidelines, adequate access to both EC and abortion, are highlighted as important aspects of reproductive health [9, 10].

The last decade has shown a decline in registered abortion care providers throughout Germany, with approximately 1100 registered providers in 2022, compared to 2030 registrations in 2003, the majority of these providers being gynaecologist in medical practices [11]. It is important to note, that according to section 12 of the ‘Pregnancy Conflict Act’, doctors are not obligated to partake in a pregnancy termination unless there is a risk to the pregnant person’s life or health [4]. According to the German Federal Statistical Office, which records all performed abortions, the number of procedures has not shown a significant decline during this same period [1]. A list of abortion providers, issued by the Federal Medical Association, shows a notable underrepresentation of medical practices in the Bavarian and Rhineland Palatinate regions [12], meaning women from these regions have to travel further to access abortion. This decline has often been associated with the legal and social status of abortion in Germany (§ 219a and § 218) [13, 14].

The legal status of abortion has been politically debated in the last decade, with many centre/left parties supporting the regulation of abortion outside of the German penal code, i.e. the decriminalisation of abortion in Germany. The current government led by the Christian Democratic Union (04.25) does not foresee a change in the law [15].

A deficit of teaching on reproductive health topics, particularly abortion care at medical schools, has also been discussed in relation to the shortages of abortion providers [16, 17]. Very little research is available on the curriculum and how it can relate to the number of abortion providers.

Therefore, the aim of this study was to:

(i) provide insight into the current content of the curriculum that is offered on both EC and abortion; (ii) assess students’ knowledge of EC and abortion; (iii) create a better understanding the extent to which medical studies shape medical students’ knowledge; and (iv) explore students’ willingness to provide abortions in their future careers.

## Materials and methods

### Survey development and structure

To better understand current attitudes towards emergency contraception and abortion, at German medical schools, an original survey was developed with SoSciSurvey, for the purpose of this study [18]. The survey addressed medical students across all semesters. No suitable validated survey, exploring the topics in this study was found in current literature. Questionnaires from similar studies that framed the opinions of medical students and assessed the curriculum were adapted and additional questions specific to the German legal and health systems were developed [19, 20]. The questionnaire was pre-tested by 16 medical students. As the survey covered two main topics, abortion and emergency contraception, it consisted of two parts employing the same question types to individually assess: personal experience, knowledge, attitude towards emergency contraception and abortion, and the teaching of these two topics. The final survey contained 70 items and was distributed in German language. The final questionnaire can be found in the additional supplementary material, in both English and German.

Students’ knowledge was assessed using six multiple-choice questions per topic, in line with the use of multiple-choice testing being the most common method in German medical schools. The questions were chosen based on the curriculum and the examination set by the Institute for Medical and Pharmaceutical Examination Questions (IMPP) and the National Competence-Based Catalogue of Learning Objectives in Medicine (NKLM) [21, 22].

Students’ attitudes and opinions were evaluated using a 4-point Likert Scale (agree – somewhat agree – somewhat disagree- disagree) Students evaluated four statements on emergency contraception and eleven statements on abortion that addressed the current laws and regulations, access to medical care, ethics, and the social climate in Germany.

The curriculum at the individual faculties was assessed using questions relating to the students´ subjective experiences, the first assessing the students’ satisfaction with the curriculum, the second and third assessing the contents of the curriculum. A 5-point Likert-Scale assessing the scope of the curriculum offered, enabling students to evaluate whether topics had been taught sufficiently, somewhat sufficiently, somewhat insufficiently, insufficiently, or not (yet) at all, from their perspective. A multiple-choice question allowed students to evaluate the practical experience (i.e. clinical electives, hospital rotations) offered within the curriculum. This section was adapted from a study by Cohen et al. ‘What should medical students be taught about abortion? An evaluation of student attitudes towards their abortion teaching and their future involvement in abortion care’ from 2021 [19].

The section “Abortion healthcare as a practising physician” assessed students’ willingness, concerns and attitudes towards providing abortions in their future careers. Willingness to perform an abortion was assessed through a yes or no question. Using a 4-point Likert Scale, students evaluated six statements, five of which began with “I would be afraid…”. These statements addressed anti-choice movements, laws and policies, as well as social acceptance and reflect situations abortion care providers in Germany have been confronted with.

### Data collection and inclusion

A pre-test was performed by 16 medical students, with small changes being made to the structure of the questions to ensure intelligibility. Data was collected between 11.06.2022 and 30.07.2022. The link for the questionnaire was distributed online amongst German medical school faculties via e-mail, WhatsApp, and other social media platforms.

In total, *n* = 1260 survey responses were received. In 2022, 108.130 medical students were enrolled throughout medical faculties in Germany, 64% of whom were female [23]. Fraudulent responses were minimised by distributing the survey only through medical faculties and faculty groups. Before the commencement of the survey, a yes or no question was used to verify that the respondent was in fact an enrolled medical student at a German faculty.

All responses with at least one response outside the “*sociodemographic characteristics*” category were included in the data analysis (*n* = 1167). A total of *n* = 93 responses were excluded.

### Statistical analysis

Survey data was analysed using SAS^®^ OnDemand for Academics [24]. Frequency tables were created for each variable in the Likert scales and the questions, and the overall trends were examined. Sociodemographic characteristics were grouped and compared. The data is described in terms of mean ± SD, frequencies (number of cases) and percentages.

## Results

### Sociodemographic

The final sample consisted of *n* = 1167 responses from medical students who were currently enrolled throughout 34 different medical faculties in Germany. The majority of respondents were female (81%). Most respondents had reached the clinical stage of their studies or were in their final year (59.4%). The average respondent age was 23.2 (± 3.6) years. Groups were created based on the stage of their studies: preclinical (1st −4th semester), clinical ((5-10th semester) and final year (11th-12th semester ‘practical year’). Further details about the characteristics of the cohort can be found in Table 1.

**Table 1:** Sociodemographic characteristics of the cohort (Germany, 2022)

Characteristics	Number (*n*)	Percentage (%)
Gender identity	*n* = 1167	
Female	947	81.2%
Male	209	17.9%
Other	11	0.9%
Children	*n* = 1166	
No	1129	96.8%
Member of a religious community	*n* = 1166	
Yes	627	53.8%
Phase of studies	*n* = 1160	
Preclinical	471	40.6%
Clinical	631	54.4%
Final Year Students	58	5%

### Opinions

Respondents were asked to evaluate 4 statements on emergency contraception and 11 statements regarding abortion using a 4-point Likert scale (agree-somewhat agree - somewhat disagree - disagree), see Table 2.

**Table 2:** Medical students’ opinion on abortion and emergency contraception, based on current regulations in Germany (Germany, 2022)

	Number (*n*)	Agree	Disagree
Emergency Contraception			
The topic of emergency contraception is stigmatised in Germany	*n* = 1073	75.2%	24.8%
Emergency contraception should generally be provided free of charge in Germany	*n* = 1074	80.2%	19.8%
Access to emergency contraception methods is adequate in Germany	*n* = 1065	67,2%	32.8%
In terms of safety, the emergency contraceptive pill is equivalent to conventional contraception with the contraceptive pill or condom.	*n* = 1072	8.1%	91.9%
Abortion			
Abortion should be generally prohibited	*n* = 999	2.7%	97.3%
I consider the current time limits for an abortion in Germany to be appropriate	*n* = 992	57.5%	42.5%
I find mandatory counselling (conflict counselling) prior to an abortion to be appropriate	*n* = 998	76.5%	23.5%
I find a waiting period between counselling and an abortion to be appropriate	*n* = 998	77.6%	23.5%
Abortion should be free of charge regardless of income	*n* = 999	87%	13%
Access to abortion is sufficient in Germany	*n* = 999	23.2%	76.8%
Specialists should be able to provide information regarding abortion on their practice website	*n* = 998	96.7%	3.3%
The legal regulations on abortion should remain in the penal code	*n* = 990	31.1%	68.9%

### Curriculum

#### Curriculum satisfaction towards emergency contraception

**Fig. 1:**
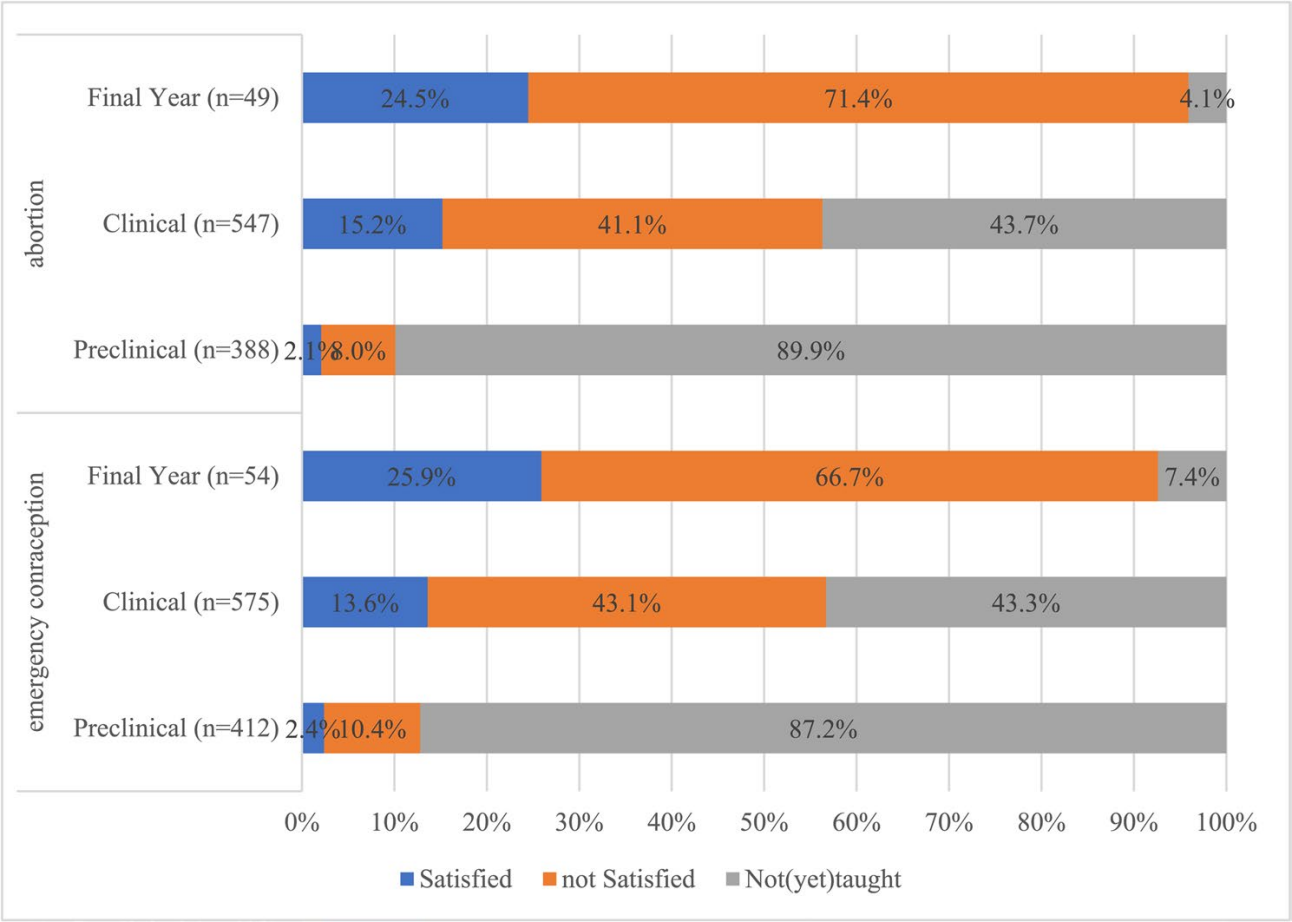
Satisfaction with the reproductive health curriculum towards emergency contraception and abortion (Germany, 2022)

When questioned whether students were satisfied with the curriculum on emergency contraception, only 9.7% of students were satisfied with the curriculum. 58.9% of respondents claim that emergency contraception was not (yet) part of the curriculum, 41% of which were in the clinical or final year stage of their studies. 31.5% of all respondents were not satisfied with the curriculum on emergency contraception. 43.3% of students in their clinical phase (*n* = 575) and 7.4% of students in their final year of studies (*n* = 54) reported to have not been taught about emergency contraception. Only 25.9% of students in their final year were satisfied with the curriculum offered on emergency contraception (see Fig. 1).

#### Curriculum satisfaction towards abortion

When questioned whether students were satisfied with their medical school’s curriculum on abortion (*n* = 989), only 10.41% of students were satisfied with the curriculum, with 29.6% of respondents reporting, that they were not satisfied with the curriculum on abortion. 60% of respondents claimed that abortion had not (yet) been taught as part of the curriculum, of which 40.5% had surpassed the preclinical stage of their studies. In their final year of studies, 71.4% of students were not satisfied with their studies on abortion, and 4.08% of students in their final year (*n* = 49) had not been taught about abortion at all (see Fig. 1).

#### Abortion and emergency contraception curriculum opportunities

**Fig. 2:**
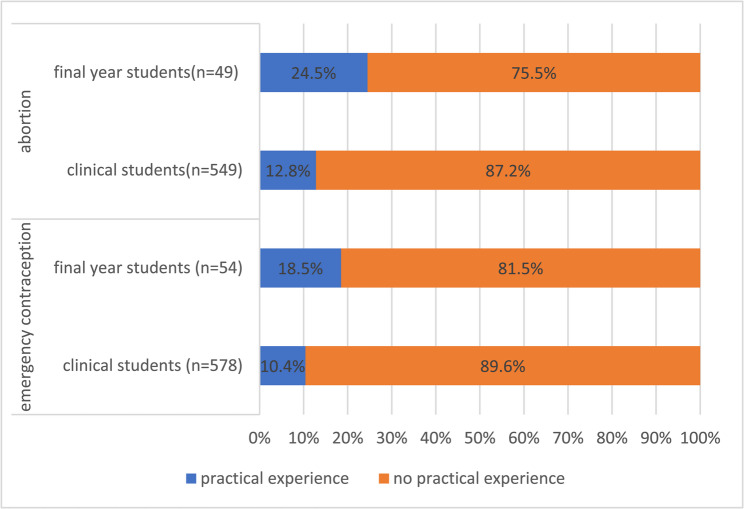
Practical experience opportunities within the curriculum at medical schools in evaluated by clinical and final year medical students in Germany (2022)

Table 3 shows the clinical and final year students’ evaluation of the content offered within their curriculum. Preclinical students’ responses are not presented in this section, as abortion and emergency contraception, are topics that are predominantly part of the clinical curriculum in Germany. Clinical experience, and thus the clinical curriculum (i.e. clinical electives, hospital rotations) starts after the 4th semester. When asked about the clinical experience offered by the curriculum, 81.5% of final year students had not received any clinical hands-on experience or training towards emergency contraception and 75.5% had not received any clinical experience in the field of abortion healthcare. Further results from students can be found in Fig. 2.

**Table 3:** Contents of the curriculum evaluated by clinical and final year medical students (Germany, 2022) The following topics were taught sufficiently during my studies:

	Number (*n*)	Agree	Disagree	Not taught
The methods of emergency contraception	*n* = 628	22.6%	35.5%	41.9%
The pharmacological effects and use of the emergency contraceptive pill	*n* = 628	33.8%	32.5%	33.8%
Indication, effect, and insertion of copper IUDs	*n* = 627	10.4%	42.7%	46.9%
The legal status of abortion in Germany	*n* = 595	40.8%	21.5%	37.7%
The ethical and moral principles of abortion in Germany	*n* = 595	37.8%	28.6%	33.6%
The various indications for abortion in Germany	*n* = 594	38.2%	22.9%	38.9%
The different methods of performing an abortion used in Germany	*n* = 594	14.5%	39.7%	45.8%

### Sources of knowledge amongst students

Table 4 shows the students’ sources of knowledge towards abortion and EC. Respondents were asked to evaluate the impact of their studies, media and personal experience or research towards their acquired knowledge regarding EC and abortion. Students were able to select multiple answers.

**Table 4:** Sources of knowledge towards emergency contraception and abortion selected by medical students (Germany,2022)

	Emergency Contraception *n* = 1051		Abortion *n* = 993		
	Number (n)	percentage (%)		Number (n)	percentage (%)
University Studies	227	21.6%		318	32%
Media	285	27.1%		414	41.8%
Personal Experience	406	38.6%		187	18.9%
Personal Research	582	55.4%		575	57.9%
Friends and Family	299	28.5%		249	25.1%

### Future perspectives on abortion healthcare

Respondents were questioned on their general willingness to offer abortion healthcare as doctors, using a yes or no option. 82.4% of respondents, reported their general willingness to provide abortion services in their future career. Furthermore, respondents were asked to respond to statements such as “I would be afraid of…if I were a doctor providing abortion” using a 4-point Likert Scale. These statements addressed anti-choice movements, laws and policies, and social acceptance. Almost half of the respondents (48.8%, *n* = 981) agreed that they would be afraid of anti-abortion activists, and 45% agreed that they would be afraid of legal prosecution. 65% of respondents (*n* = 982) did not that agree they would fear discrimination and 81.1% (*n* = 981) did not fear negative perception amongst friends and family.

## Discussion

### Emergency contraception (EC)

Emergency contraception remains an important topic for physicians as they still play an important role in advising patients on EC, even after the switch to over the counter (OTC) status in Germany in 2015 [8]. Close to half of EC users still received a prescription from a physician in 2019 [7]. The perceived stigmatisation of EC by 75.2% of respondents stands out in the results of this study. The relatively late switch of EC to OTC in Germany, was highly debated at the time. Many physicians opposed the change, highlighting fears of misuse and the necessity of medical counselling [25]. According to research, stigma surrounding the use of EC also still exists. A 2018 survey by ‘Profamilia’, a reproductive health service, reported that many users had experienced indiscretion, stigmatisation, lecturing and blame from pharmacists when purchasing EC [26].

When evaluating the curriculum, the topic of EC received a lower satisfaction rate than abortion, with little to no practical teaching being offered. When asked about their main source of knowledge, most students named their personal experience, followed by their studies. This suggests that students have used or personally researched EC independently of their studies. A German Federal Centre for Health Education survey states that 27% of sexually active women aged between 18 and 25 have used EC [27].

### Abortion

The respondents to this study generally hold a positive view toward the legality of abortion and support the removal of abortion from the penal code. Previous research showed, that although negative attitudes towards abortion have increased since the reunification of Germany, women and/or respondents with a higher education degree are less likely to hold restrictive views on abortion [28]. Other studies have surveyed medical students on the legal status of abortion in their country of study. For example, 83% of British medical students surveyed in 2021 described their attitude as ‘pro-choice’. In a 2014 study, 55% of Irish medical students believed that abortion, which at the time was still criminalised, should be legally available on request [29]. Abortion has since been legalised in Ireland. In our study, 69% of students supported abortion being regulated outside of the German penal code.

When asked about specific aspects of the current legislation, our respondents were more likely to endorse mandatory counselling and a mandatory waiting period (MWP) before an abortion. These requirements are not recommended by the WHO and have been refuted by medical publications as they represent significant barriers to abortion care, they are however required when seeking out abortion care in Germany under article 218a [9, 30, 31].

The results of this study reveal that there is little teaching available towards abortion at medical schools throughout Germany, as evidenced by the fact that students cited their studies as only the third main source of knowledge on this topic, after personal research and the media. When abortion is taught at German medical faculties, the results suggest a larger focus on ethics and law rather than on medical aspects. It is important to note that medical learning objectives towards abortion and EC exist and are included in the catalogue of learning objectives for medical students published by the Institute for Medical and Pharmaceutical Examination Questions (IMPP) [21].

This study does not directly provide specific insight into why abortion and EC are not taught more thoroughly. Further research aimed at education providers would be needed to understand the barriers to reproductive health education German medical schools.

A global survey published in 2022, investigating the inclusion of sexual and reproductive health rights (SRHR) topics in medical schools found that higher SRHR content could be positively associated with low abortion restrictions in that country [32]. Rennison et al. found that existing barriers to abortion education in the United Kingdom included difficulties in accessing clinical placements, a lack of curriculum time and the sensitive nature of abortion as a topic [33]. There is an abundance of socio-political barriers to accessing abortion services in Germany, which, when put in context, may be associated with difficulties integrating abortion into the national medical school curriculum [34, 35].

As abortion is a common medical procedure, even if they do not provide abortion healthcare themselves, it is likely that (future) doctors will be confronted with the procedure during their career, which underlines the importance of teaching the topic despite its complicated legal status. This creates a direct conflict for medical educators as there is a discrepancy between Germany’s current abortion laws and the medical standards recommended the WHO, which highlights the need for medically based teaching to provide equitable and safe abortions [9].

The limited number of physicians offering abortion as a medical procedure simultaneously means that less clinical teaching/learning opportunities are available to students. In Germany, most abortions performed before the 14th week of gestation are offered in outpatient settings [1, 36]. Consequently, medical students are less likely to encounter the procedure within a university hospital setting.

Additionally, in a 2019 publication, Hänel, a German abortion provider, describes an information deficit among educators, caused by fear of criminal charges under § 219a for publicly discussing abortion [16]. As mentioned in the introduction, this law has since been abolished. This study is unable to directly assess the impact of the abolishment and whether it will lead to improvements to the curriculum in the future.

It is also important to note that approximately a third of all hospitals in Germany are run by denominational institutions [37]. The Roman-Catholic and Protestant branches of the church hold restrictive views on abortion and EC and rarely offer abortion care within their institutions [38, 39]. Furthermore, personal religious beliefs or fear of stigmatisation through religious family members can influence doctors who claim conscience objection to providing an abortion, according to a study by Baier et al., questioning doctors and medical students about personal barriers to abortion care in Germany [40]. Our results showed that 18.9% of respondents would fear negative perception amongst family members and friends, although our respondents were not specifically questioned on the influence of religious beliefs.

In a 2020 correspondence article published in *The Lancet*, the current decline in access to abortion in Germany is described as a threat to health equity [41]. Although we did not question our respondents on methods and gestational age, our respondents showed a high general willingness to be involved in abortion care (82,4%). A 2021 study questioning medical students in the UK, where abortion on request is legal, found, that 73% of respondents were willing to provide medication abortion and 65% were willing to provide surgical abortion at < 12 weeks of gestation. This criterion reflects the current gestational limits in abortion law in Germany, providing a basis for comparison between the two groups [19]. It is important to note that, according to our results, a minimal number of students have seen or have assisted in the procedure of an abortion and that this number does not correspond to the number of students who will go on to provide abortion care.

The possibility of being prosecuted for providing abortion care or of being confronted by anti-abortion activists present barriers for abortion care providers. Under § 219a, physicians have been prosecuted for providing factual information on their websites, with initial complaints to the police originating from anti-abortion activists [42]. This received a lot of publicity at the time and intensified the political debate on whether the law, which has now been abolished, was justified in describing medial information on abortion methods as advertising.

Physicians are still subject to abortion-related stigma in Germany [17, 34]. Anti-abortion activists play a significant role in the chastisement of physicians, with many reports of public harassment towards doctors. This has resulted in the president of the German Federal Medical Association demanding better protection for physicians with an amendment to the ‘pregnancy conflict act’ being made, which now protects counselling centres and medical institutions from third-party harassment [4].

## Study strengths and limitations

We were able to reach a variability of medical students,’ from a range of faculties, although female respondents were slightly overrepresented, and not all faculties were reached. As this survey was a voluntary online survey on a sensitive topic, it is subject to a wide range of selection bias from both pro- and anti-abortion respondents. We did not differentiate between medication and procedural abortion, which is a further limitation. The evaluation of the medical school curriculum was made subjectively by respondents and offers a one-sided insight into the curriculum.

## Conclusion

Medical students in Germany hold a generally positive attitude towards abortion and EC. Personal attitudes towards abortion can influence doctors providing abortion; however, our respondents demonstrate a strong conviction towards better access to abortion and the decriminalisation of abortion. Legal status, bureaucratic barriers or stigmatization could be stronger influences on doctors’ decision to conscientiously object to providing an abortion.

We conclude that there is a gap in the reproductive health curriculum at German medical schools. Abortion and EC remain stigmatised topics in Germany, and the known political and legal barriers affecting access to abortion care could be impacting the quality of education offered. Further research into the curriculum from the educators’ perspectives could support this conclusion and help to understand where these education gaps originate. Moreover, further research into the decline in abortion providers in Germany is necessary, to understand why doctors choose not to be abortion providers.

Based on global normative work and our findings, we conclude that a non-biased, evidence-based approach to reproductive health topics at medical schools should become standard practice, improving the quality of education offered and supporting future doctors in their choice to provide abortion care.

## Supplementary Information

Below is the link to the electronic supplementary material.

Supplementary Material 1

Supplementary Material 2

## Data Availability

Data can be made available upon request from the corresponding author at [cecilia.rees@rwth-aachen.de](mailto: cecilia.rees@rwth-aachen.de).

## Abbreviations

EC: emergency contraception
MWP: mandatory waiting period
SRHR: sexual and reproductive health rights
OTC: over the counter
