# Supplementary material for: Evaluation of the reproductive health curriculum at medical schools in Germany: an insight into medical students’ knowledge and opinion towards emergency contraception and abortion - a cross-sectional study
Source: BMC Public Health. 2025 Sep 24;25:3056. doi: 10.1186/s12889-025-24492-4 (PMC12462158; doi:10.1186/s12889-025-24492-4)
Supplement: Supplementary file 1 — Supplementary Material 1 [file 12889_2025_24492_MOESM1_ESM.pdf]

soSci
oFb - der onlineFragebogen

ReproduktiveGesundheit22 → base 14.09.2022, 19:39

Seite 01

Liebe Studierende der Medizin,

mein Name ist Cecilia Rees, ich bin Medizinstudentin an der RWTH Aachen und verfasse derzeit meine Doktorarbeit im Themenbereich Reproduktive Gesundheit. Hierzu würde ich gerne mittels dieser Umfrage, die Einstellung und den Wissensstand von Medizinstudierenden an verschiedenen Universitäten in Deutschland zu den Themen Notfallkontrazeption und Schwangerschaftsabbruch erheben. Genauso möchte ich das Lehrangebot zu diesen Themen evaluieren.

Das Ausfüllen der Befragung dauert **maximal 15 Minuten**. Für den Erfolg der Studie sollten die Angaben wahrheitsgemäß und möglichst vollständig sein.

Die Teilnahme an der Umfrage ist **freiwillig**. Ihr könnt den Fragebogen jederzeit abbrechen. Alle Daten werden **anonym** erhoben, sie können eurer Person nicht zugeordnet werden und werden streng vertraulich behandelt.

Vielen Dank für eure Teilnahme.

Cecilia Rees

Bei Fragen oder Rückmeldung bitte wenden an: cecilia.rees@rwth-aachen.de

Seite 02

1. Studierst du zurzeit Humanmedizin an einer deutschen Universität?

- ◯ Ja
- ◯ Nein

Teil Soziodemographie

Folgende Fragen dienen der Einstufung deiner Person.

2. Alter

[ ] Jahre (bitte eintragen)

3. Geschlecht

- ◯ weiblich
- ◯ männlich
- ◯ divers

4. Hast du Kinder?

- ◯ Ja
- ◯ Nein

5. Zu welcher der folgenden Religionen fühlst du dich zugehörig?

- ◯ Keine
- ◯ Christentum
- ◯ Judentum
- ◯ Islam
- ◯ Hinduismus
- ◯ Buddhismus
- ◯ Sonstige [ ]

6. An welcher deutschen Universität studierst du?

Anordnung alphabetisch nach Städten

[Bitte auswählen]

7. In welchem Fachsemester studierst du?

Bitte Semesterzahl eingeben

[ ] Semester

8. Welche der folgenden klinischen Fachgebiete interessieren dich am meisten?

Mehrfachwauswahl möglich

- ☐ Anästhesiologie
- ☐ Augenheilkunde
- ☐ Radiologie (inklusive Nuklearmedizin)
- ☐ Chirurgie (Allgemeinchirurgie, Herz- oder Gefäßchirurgie, Neurochirurgie, Mund-Kiefer- Gesichtschirurgie, usw.)
- ☐ Dermatologie
- ☐ Gynäkologie und Geburtshilfe
- ☐ Innere Medizin
- ☐ Kinder- und Jugendmedizin
- ☐ Neurologie
- ☐ Psychiatrie (inkl. Psychosomatik)
- ☐ Urologie

Seite 04

Der folgende Teil des Fragebogens beschäftigt sich mit dem Thema **Notfallkontrazeption**

Hinweis:

Diese Umfrage ist für alle Studierende der Medizin freigeschaltet, hierdurch werden manche Fragen über deinen Wissensstand hinausgehen. Ich würde dich trotzdem bitten, den Fragebogen für den Erfolg der Studie **ehrlich und ohne Hilfe** auszufüllen.

Seite 05

Persönliche Erfahrungen

9. Ich habe selbst schon mal eine Form der Notfallkontrazeption angewendet.

- ◯ Ja
- ◯ Nein
- ◯ trifft nicht auf mich zu

10. Mein*e Partner*in hat schon mal eine Form der Notfallkontrazeption angewendet.

- ◯ Ja
- ◯ Nein
- ◯ trifft nicht auf mich zu

Wissensfragen Notfallkontrazeption

11. Welches der folgenden Notfallkontrazeptiva hat die höchste Wirksamkeit ?

- ◯ Postkoitale Kontrazeptiva („Pille danach")
- ◯ Kupferspirale

12. In welcher/n Zyklusphase/n „wirkt" die Pille danach?

- ☐ Follikelphase (vor dem Eisprung)
- ☐ Ovulationsphase (während des Eisprungs)
- ☐ Lutealphase (nach dem Eisprung)

13. Welche der folgenden Wirkungsweisen trifft auf die Kupferspirale zu?

Mehrfachauswahl möglich

- ☐ Verhinderung der Einnistung einer befruchteten Eizelle in die Gebärmutter (Implantation)
- ☐ Verzögerung des LH Peaks und somit der Ovulation
- ☐ Hemmung der Ovulation für den kompletten Zyklus
- ☐ Veränderung des Zervixschleims zur Spermienabwehr

14. Die Kupferspirale kann als Notfallkontrazeptivum bis zu welchem Zeitpunkt spätestens eingesetzt werden?

- ◯ bis zu 24h nach dem Geschlechtsverkehr
- ◯ bis zu 48h nach dem Geschlechtsverkehr
- ◯ bis zu 72h nach dem Geschlechtsverkehr
- ◯ bis zu 4 Tage nach dem Geschlechtsverkehr
- ◯ bis zu 5 Tage nach dem Geschlechtsverkehr

15. Die „Pille danach" enthält die gleichen Wirkstoffe wie die „Abbruchpille" und wirkt somit abortiv.

- ◯ Ja
- ◯ Nein

16. Die „Pille danach" ist ein verschreibungspflichtiges Medikament.

- ◯ Ja
- ◯ Nein

Einstellung zu Notfallkontrazeption

17. Einstellung zu Notfallkontrazeption

	Stimme zu	Stimme eher zu	Stimmer eher nicht zu	Stimme nicht zu
Notfallkontrazeption ist in Deutschland ein stigmatisiertes Thema.	○	○	○	○
Notfallkontrazeption sollte in Deutschland grundsätzlich kostenfrei zur Verfügung gestellt werden.	○	○	○	○
Der Zugang zur Notfallkontrazeption ist in Deutschland angemessen.	○	○	○	○
Bezogen auf die Sicheheit, ist die „Pille danach“ der gängigen Empfängnisverhütung durch die Pille oder das Kondom gleichzusetzen.	○	○	○	○

Lehre an deiner Universität

18. Lehrangebot

Ich bin mit dem Lehrangebot zum Thema Notfallkontrazeption an meiner Universität zufrieden.

Stimme zu	Stimmer eher zu	Stimme eher nicht zu	Stimme nicht zu	*wurde (noch) nicht gelehrt*

19. Folgende Themen wurden in meinem Studium ausreichend besprochen:

Möglichkeiten und Methoden der Notfallkontrazeption

stimme zu	stimmer eher zu	Stimme eher nicht zu	Stimme nicht zu	*wurde (noch) nicht besprochen*

pharmakologische Wirkungsweise und Anwendung der „Pille danach“

stimme zu	stimmer eher zu	Stimme eher nicht zu	Stimme nicht zu	*wurde (noch) nicht besprochen*

Indikation, Wirkung und praktisches Vorgehen bei Kupferspiralen

stimme zu	stimmer eher zu	Stimme eher nicht zu	Stimme nicht zu	*wurde (noch) nicht besprochen*

20. Ich habe folgende praktische Erfahrungen schon im Rahmen meines Studiums gemacht:
Wähle bitte Zutreffendes aus

- ☐ das Beobachten der Einsetzung einer Spirale am Patienten
- ☐ das Beobachten der Einsetzung einer Spirale am Modell
- ☐ das Durchführen der Einsetzung einer Spirale am Modell
- ☐ das Beobachten einer Beratung/Verordnung von Notfallkontrazeption
- ☐ keine praktischen Erfahrungen gemacht

21. Ich fühle mich in der Lage,

	stimme zu	stimmer eher zu	Stimme eher nicht zu	Stimme nicht zu
die verschiedenen Methoden der Notfallkontrazeption zu erläutern.	◯	◯	◯	◯
die Wirkung und Nebenwirkungen der „Pille danach“ zu erläutern.	◯	◯	◯	◯
die Wirkung und Nebenwirkungen der Kupferspirale zu erläutern.	◯	◯	◯	◯
das praktische Vorgehen bei der Einnahme der „Pille danach“ zu erläutern.	◯	◯	◯	◯
das praktische Vorgehen bei dem Einsetzten einer Kupferspirale zu erläutern.	◯	◯	◯	◯

22. Mein Wissen zum Thema Notfallkontrazeption habe ich vor allem erworben über:

Wähle bitte Zutreffendes aus

- ☐ Studium
- ☐ Medien
- ☐ eigene Erfahrungen
- ☐ eigene Recherche
- ☐ Freunde und Familie
- ☐ Ich habe keine ausreichende Kenntnisse zu diesem Thema

Seite 09

Der folgende Teil des Fragebogens beschäftigt sich mit dem Thema **Schwangerschaftsabbruch**

Hinweis:

Diese Umfrage ist für alle Studierende der Medizin freigeschaltet, hierdurch werden manche Fragen über deinen Wissensstand hinausgehen. Ich würde dich trotzdem bitten, den Fragebogen für den Erfolg der Studie **ehrlich und ohne Hilfe** auszufüllen.

Persönliche Erfahrungen

23. Ich habe schon einmal eine eigene Schwangerschaft abgebrochen.

- ◯ Ja
- ◯ Nein
- ◯ trifft nicht auf mich zu

24. Mein*e Partner*in hat schonmal eine Schwangerschaft abgebrochen.

- ◯ Ja
- ◯ Nein
- ◯ trifft nicht auf mich zu

Wissensfragen SSAB

BITTE LESEN : Schwangerschaftswochen

Zur Datierung einer Schwangerschaft gibt es folgende Möglichkeiten:

Menstruationsalter (**post menstruationem**) in Schwangerschaftswochen (SSW pm)

ab dem ersten Tag der letzten Menstruation (üblicherweise in der Gynäkologie angewendet)

Ovulationsalter (**post conceptionem**) in Entwicklungswochen (SSW pc)

ab dem letzten Eisprung (tatsächliches Schwangerschaftsalter)

In diesem Fragebogen wird die Datierung post menstruationem angewendet.

25. Die Fristenregelung erlaubt den Schwangerschaftsabbruch nach Beratungsregelung oder nach kriminologischer Indikation bis zu welchem Zeitpunkt?

- ◯ 12 SSW
- ◯ 14 SSW
- ◯ 16 SSW
- ◯ 18 SSW
- ◯ 20 SSW
- ◯ Immer

26. Die Fristenregelung erlaubt den Schwangerschaftsabbruch bei medizinischer Indikation bis zu welchem Zeitpunkt?

- ◯ 12 SSW
- ◯ 14 SSW
- ◯ 16 SSW
- ◯ 18 SSW
- ◯ 20 SSW
- ◯ Immer

27. Wie lange beträgt die vorgeschriebene Wartezeit zwischen verpflichtender Schwangerschaftskonfliktberatung und dem Schwangerschaftsabbruch (nach Beratungsregelung)?

- ◯ Es gibt keine Wartezeit
- ◯ 2 Tage
- ◯ 3 Tage
- ◯ 5 Tage
- ◯ 7 Tage

28. Das operative Standardverfahren für einen Schwangerschaftsabbruch in der 11 SSW in Deutschland ist

- ◯ die manuelle Vakuumaspiration
- ◯ die elektrische Vakuumaspiration
- ◯ die Ausschabung/Kürretage
- ◯ die fraktionierte Abrasio

29. Der medikamentöse Schwangerschaftsabbruch (Progesteronrezeptor-Antagonist (Mifepriston) mit nachfolgender Weheninduktion (Prostaglandin)) ist zugelassen bis zur einschließlich

- ◯ 9 SSW
- ◯ 10 SSW
- ◯ 11 SSW
- ◯ 12 SSW
- ◯ 14 SSW

30. Ein Schwangerschaftsabbruch erhöht das Risiko für zukünftige Schwangerschaftskomplikationen.

- ◯ Ja
- ◯ Nein

Einstellung zum Schwangerschaftsabbruch

31. Persönliche Einstellung zum Schwangerschaftsabbruch

	stimme zu	Stimme eher zu	Stimme eher nicht zu	Stimme nicht zu
Ein Schwangerschaftsabbruch sollte prinzipiell verboten sein.	○	○	○	○
Ein Schwangerschaftsabbruch sollte bei einer gesundheitlichen Beeinträchtigung der Mutter (medizinische Indikation) erlaubt sein.	○	○	○	○
Ein Schwangerschaftsabbruch sollte nach Vergewaltigung oder Kindesmissbrauch (kriminologische Indikation) erlaubt sein.	○	○	○	○
Ein Schwangerschaftsabbruch sollte bei ausdrücklichem Wunsch der Schwangeren (Beratungsregel) erlaubt sein.	○	○	○	○
Die aktuellen Fristen für einen Schwangerschafsabbruch in Deutschland halte ich für angemessen.	○	○	○	○
Eine verpflichtende Schwangerschaftskonfliktberatung halte ich für sinnvoll.	○	○	○	○
Eine Wartezeit zwischen Beratung und Eingriff halte ich für sinnvoll.	○	○	○	○
Ein Schwangerschaftsabbruch sollte unabhängig vom Einkommen kostenfrei sein.	○	○	○	○
Der Zugang zum Schwangerschaftsabbruch ist in Deutschland angemessen.	○	○	○	○
Fachärzte sollten Informationen zum Schwangerschaftsabbruch auf ihrer Praxis-Webseite zur Verfügung stellen dürfen.	○	○	○	○
Die rechtlichen Regelungen zum Schwangerschaftsabbruch sollten weiterhin im Strafgesetzbuch niedergeschrieben sein.	○	○	○	○

Lehre an deiner Universität

32. Lehrangebot Schwangerschaftsabbruch

Ich bin mit dem Lehrangebot zum Thema Schwangerschaftsabbruch an meiner Universität zufrieden.

Stimme zu	Stimme eher zu	Stimme eher nicht zu	Stimme nicht zu	*wurde (noch) nicht gelehrt*

33. Folgende Themen wurden in meinem Studium ausreichend besprochen:

rechtliche Grundlagen des Schwangerschaftsabbruchs in Deutschland

stimme zu	stimmer eher zu	Stimme eher nicht zu	Stimme nicht zu	*wurde (noch) nicht besprochen*

ethisch-moralische Grundlagen des Schwangerschaftsabbruchs in Deutschland

stimme zu	stimmer eher zu	Stimme eher nicht zu	Stimme nicht zu	*wurde (noch) nicht besprochen*

die verschiedenen Indikationen des Schwangerschaftsabbruchs in Deutschland

stimme zu	stimmer eher zu	Stimme eher nicht zu	Stimme nicht zu	*wurde (noch) nicht besprochen*

die verschiedenen Methoden des Schwangerschaftsabbruchs in Deutschland

stimme zu	stimmer eher zu	Stimme eher nicht zu	Stimme nicht zu	*wurde (noch) nicht besprochen*

34. Ich habe folgende praktische Erfahrungen im Rahmen meines Studiums gemacht:

Wähle bitte Zutreffendes aus

- [ ] einen operativen Schwangerschaftsabbruch beobachtet
- [ ] einen medikamentösen Schwangerschaftsabbruch beobachtet
- [ ] eine Schwangerschaftskonfliktberatung beobachtet
- [ ] keine praktischen Erfahrungen gemacht

35. Ich fühle mich in der Lage,

	stimme zu	stimmer eher zu	Stimme eher nicht zu	Stimme nicht zu
die rechtliche Grundlagen des Schwangerschaftsabbruch zu erläutern.	○	○	○	○
die verschiedenen Indikationen für einen Schwangerschaftsabbruch zu prüfen.	○	○	○	○
das Verfahren des medikamentösen Schwangerschaftsabbruchs zu erläutern.	○	○	○	○
das Verfahren des operativen Schwangerschaftsabbruchs zu erläutern.	○	○	○	○
eine Schwangerschaftskonfliktberatung durchzuführen.	○	○	○	○

36. Mein Wissen zum Thema Schwangerschaftsabbruch habe ich vor allem erworben über:

Wähle bitte Zutreffendes aus

- ☐ Studium
- ☐ Medien
- ☐ eigene Erfahrungen
- ☐ eigene Recherche
- ☐ Freunde und Familie
- ☐ Ich habe keine ausreichende Kenntnisse zu diesem Thema

Der Schwangerschaftsabbruch aus ärztlicher Perspektive

37. Ich könnte mir prinzipiell vorstellen, als Arzt oder Ärztin einen Schwangerschaftsabbruch anzubieten.

- ◯ Ja
- ◯ Nein

	Stimme zu	Stimme eher zu	Stimmer eher nicht zu	Stimme nicht zu
Ich hätte Angst, diskriminiert zu werden, wenn ich einen Schwangerschaftsabbruch anbieten würde.	◯	◯	◯	◯
Ich hätte Angst, von Freund*innen oder Familie negativ beurteilt zu werden, wenn ich einen Schwangerschaftsabbruch anbieten würde.	◯	◯	◯	◯
Ich hätte Angst, rechtlich verfolgt zu werden, wenn ich einen Schwangerschaftsabbruch anbieten würde.	◯	◯	◯	◯
Ich hätte Angst vor Abtreibungsgegner*innen, wenn ich einen Schwangerschaftsabbruch anbieten würde.	◯	◯	◯	◯
Ich hätte Angst, Patient*innen zu verlieren, wenn ich einen Schwangerschaftsabbruch anbieten würde.	◯	◯	◯	◯
Medizinisches Personal sollte verpflichtet sein, Patient*innen an eine ausgebildete Fachkraft zu verweisen, wenn sie selbst keinen Schwangerschaftsabbruch durchführen wollen.	◯	◯	◯	◯

Letzte Seite

Vielen Dank für Ihre Teilnahme!

Wir möchten uns ganz herzlich für Ihre Mithilfe bedanken.

Ihre Antworten wurden gespeichert, Sie können das Browser-Fenster nun schließen.

Möchten Sie in Zukunft an interessanten und spannenden Online-Befragungen teilnehmen?

Wir würden uns sehr freuen, wenn Sie Ihre E-Mail-Adresse für das SoSci Panel anmelden und damit wissenschaftliche Forschungsprojekte unterstützen.

E-Mail: ____________ [Am Panel teilnehmen]

Die Teilnahme am SoSci Panel ist freiwillig, unverbindlich und kann jederzeit widerrufen werden. Das SoSci Panel speichert Ihre E-Mail-Adresse nicht ohne Ihr Einverständnis, sendet Ihnen keine Werbung und gibt Ihre E-Mail-Adresse nicht an Dritte weiter.

Sie können das Browserfenster selbstverständlich auch schließen, ohne am SoSci Panel teilzunehmen.

Cecilia Rees, Uniklinik RWTH Aachen – 2022
