# Supplementary material for: Evaluation of the reproductive health curriculum at medical schools in Germany: an insight into medical students’ knowledge and opinion towards emergency contraception and abortion - a cross-sectional study
Source: BMC Public Health. 2025 Sep 24;25:3056. doi: 10.1186/s12889-025-24492-4 (PMC12462158; doi:10.1186/s12889-025-24492-4)
Supplement: Supplementary file 2 — Supplementary Material 2 [file 12889_2025_24492_MOESM2_ESM.docx]

**Reproductive Health Survey, Germany, 2022
Authors: Cecilia Rees, Andrea Kafie**

**Translated from original (German Language)**

**Page 1**

Dear medical students,

My name is Cecilia Rees, and I am a medical student at RWTH Aachen University currently working on my doctoral thesis in the field of reproductive health. Through this survey, I would like to gather information on the attitudes and knowledge of medical students at various universities in Germany regarding emergency contraception and abortion. Additionally, I aim to evaluate the educational curriculum offered on these topics.

Completing the survey will take **a maximum of 15 minutes**. For the success of the study, the information provided should be truthful and as complete as possible.

Participation in the survey is **voluntary**. You can stop filling out the questionnaire at any time. All data will be collected anonymously, cannot be attributed to your person, and will be treated with strict confidentiality.

Thank you for your participation.

Cecilia Rees

For questions or feedback, please contact: cecilia.rees@rwth-aachen.de

**Page 2**

1. Are you currently studying medicine at a German university?
   - Yes
   - No

**Page 3**

**Sociodemographic information**

The following questions are intended to categorize your person.

1. Age
   - ___ years (please enter)
2. Gender
   - Female
   - Male
   - Divers
3. Do you have children?
   - Yes
   - No
4. Which of the following religions do you feel affiliated with?
   - None
   - Christianity
   - Judaism
   - Islam
   - Hinduism
   - Buddhism
   - Other: ___
5. At which German university are you currently studying?
   - [Please select]
6. In which semester are you studying?

Please enter the number of semesters

- - ___ Semester

1. Which of the following clinical fields interest you the most? (Multiple selections possible)
   - Anaesthesiology
   - Ophthalmology
   - Radiology (including nuclear medicine)
   - Surgery (General Surgery, Cardiac or Vascular Surgery, Neurosurgery, Oral and Maxillofacial Surgery, etc.)
   - Dermatology
   - Gynaecology and Obstetrics
   - Internal Medicine
   - Paediatrics
   - Neurology
   - Psychiatry (including Psychosomatics)
   - Urology

**Page 4**

The following part of the questionnaire deals with **emergency contraception**.

**Note:** This survey is open to all medical students, so some questions may go beyond your knowledge. However, I would still ask you to fill out the questionnaire **honestly and without assistance** for the success of the study.

**Page 5**

**Personal Experience**

1. I have used a form of emergency contraception before.

- Yes
- No
- Not applicable

1. My partner has used a form of emergency contraception before.

- Yes
- No
- Not applicable

**Page 6**

**Knowledge Questions on Emergency Contraception**

1. Which of the following emergency contraceptives is the most effective?

- Postcoital contraceptives ("morning-after pill")
- Copper IUD

1. In which phase(s) of the cycle does the "morning-after pill" work?

- Follicular phase (before ovulation)
- Ovulation phase (during ovulation)
- Luteal phase (after ovulation)

1. Which of the following mechanisms of action apply to the copper IUD? (Multiple selections possible)

- Prevention of implantation of a fertilized egg in the uterus (implantation)
- Delay of the LH peak and thus ovulation
- Inhibition of ovulation for the entire cycle
- Alteration of cervical mucus to block sperm

1. The copper IUD can be used as an emergency contraceptive up to what point after intercourse?

- Up to 24 hours after intercourse
- Up to 48 hours after intercourse
- Up to 72 hours after intercourse
- Up to 4 days after intercourse
- Up to 5 days after intercourse

1. The "morning-after pill" contains the same active ingredients as the "abortion pill" and thus acts as an abortifacient.

- Yes
- No

1. The "morning-after pill" is a prescription medication.

- Yes
- No

**Page 7**

**Attitudes Towards Emergency Contraception**

1. Attitudes towards emergency contraception:

- Agree
- Somewhat agree
- Somewhat disagree
- Disagree

Statements:

- Emergency contraception is a stigmatized topic in Germany.
- Emergency contraception should generally be provided free of charge in Germany.
- Access to emergency contraception in Germany is adequate.
- In terms of safety, the "morning-after pill" is equivalent to conventional contraception with the pill or condom.

**Page 8**

**Education at Your University**

1. Educational curriculum offered

- Agree
- Somewhat agree
- Somewhat disagree
- Disagree
- Has not been taught (yet)

Statement:

- I am satisfied with the curriculum on emergency contraception at my university

1. The following topics were sufficiently covered in my studies:

- Agree
- Somewhat agree
- Somewhat disagree
- Disagree
- Has not been taught (yet)

Topics:

- Possibilities and methods of emergency contraception
- Pharmacological mode of action and use of the “morning after pill”
- Indication, effect and practical procedure for copper spirals

1. I have had the following practical experiences during my studies: (Select all that apply)

- Observing the insertion of an IUD in a patient
- Observing the insertion of an IUD in a model
- Performing the insertion of an IUD in a model
- Observing a consultation/prescription of emergency contraception
- No practical experience

**Personal Competence**

1. I feel able to:

- Agree
- Somewhat agree
- Somewhat disagree
- Disagree

Statements:

- Explain the various methods of emergency contraception
- Explain the effects and side effects of the "morning-after pill"
- Explain the effects and side effects of the copper IUD
- Explain the practical procedures for taking the "morning-after pill"
- Explain the practical procedures for inserting a copper IUD

1. I have acquired my knowledge on emergency contraception primarily through: (Select all that apply)

- Studies
- Media
- Personal experiences
- Personal research
- Friends and family
- I do not have sufficient knowledge on this topic

**Page 9**

The following part of the questionnaire deals **with abortion**.

**Note:** This survey is open to all medical students, so some questions may go beyond your knowledge. However, I would still ask you to fill out the questionnaire **honestly and without assistance** for the success of the study.

**Page 10**

**Personal Experiences**

1. Have you ever had an abortion yourself?

- Yes
- No
- Not applicable

1. Has your partner ever had an abortion?

- Yes
- No
- Not applicable

**Page 11**

**Knowledge Questions on Abortion**

Please read: Gestational weeks

To date a pregnancy, the following methods can be used:

- Menstrual age (post menstruationem) in gestational weeks (GW pm) from the first day of the last menstrual period (commonly used in gynaecology)
- Ovulation age (post conceptionem) in developmental weeks (GW pc) from the last ovulation (actual pregnancy age)

In this questionnaire, dating is done post menstruationem.

1. The time limit regulation allows abortion after counselling or due to criminological indication until when.

- 12^th^ week of pregnancy
- 14^th^ week of pregnancy
- 16^th^ week of pregnancy
- 18^th^ week of pregnancy
- 20^th^ week of pregnancy
- Always

1. The time limit regulation allows abortion due to medical indication until when.

- 12^th^ week of pregnancy
- 14^th^ week of pregnancy
- 16^th^ week of pregnancy
- 18^th^ week of pregnancy
- 20^th^ week of pregnancy
- Always

1. What is the required waiting period between the mandatory pregnancy ‘conflict counselling the abortion (after counselling regulation)?

- No waiting period
- 2 days
- 3 days
- 5 days
- 7 days

1. The standard operative procedure for an abortion in the 11th week of pregnancy in Germany is:

- Manual vacuum aspiration
- Electric vacuum aspiration
- Curettage
- Fractional curettage

1. The medical abortion (progesterone receptor antagonist (Mifepristone) followed by labour induction (Prostaglandin)) is approved until:

- 9^th^ week of pregnancy
- 10^th^ week of pregnancy
- 11^th^ week of pregnancy
- 12^th^ week of pregnancy
- 14^th^ week of pregnancy

1. An abortion increases the risk of future pregnancy complications.

- Yes
- No

**Page 12**

**Attitudes Towards Abortion**

1. Personal attitude towards abortion:

- Agree
- Somewhat agree
- Somewhat disagree
- Disagree

Statements:

- Abortion should be generally prohibited.
- Abortion should be allowed in case of health impairment of the mother (medical indication).
- Abortion should be allowed after rape or child abuse (criminological indication).
- Abortion should be allowed at the explicit request of the pregnant woman (counselling regulation).
- I consider the current time limits for abortion in Germany appropriate.
- I consider mandatory pregnancy ‘conflict counselling’ meaningful/sensible.
- I consider a waiting period between counselling and the procedure meaningful/sensible.
- Abortion should be free of charge regardless of income.
- Access to abortion in Germany is adequate.
- Specialists should be allowed to provide information about abortion on their medical practice website.
- The legal regulations on abortion should remain in the penal code.

**Page 13**

**Education at Your University**

1. Educational offerings

- Agree
- Somewhat agree
- Somewhat disagree
- Disagree
- Has not been taught (yet)

Statement:

I am satisfied with the curriculum on abortion at my university

1. The following topics were sufficiently covered in my studies:

- Agree
- Somewhat agree
- Somewhat disagree
- Disagree
- Has not been taught yet

Topics:

- Indication and procedure of medically induced abortion
- Indication and procedure of instrumental/operative abortion
- Legal regulations on abortion in Germany

1. I have had the following practical experiences during my studies: (Select all that apply)

- Observing an abortion
- Performing an abortion in an animal model
- No practical experience

**Personal Competence**

1. I feel able to:

- Agree
- Somewhat agree
- Somewhat disagree
- Disagree

Statements:

- Explain the various methods of abortion
- Explain the effects and side effects of medical abortion
- Explain the effects and side effects of instrumental abortion
- Explain the legal regulations on abortion in Germany

1. I have acquired my knowledge on abortion primarily through: (Select all that apply)

- Studies
- Media
- Personal experiences
- Personal research
- Friends and family
- I do not have sufficient knowledge on this topic

**Page 14**

1. I could generally imagine offering an abortion as a doctor.

- Yes
- No

1. Evaluate the following statements.

- Agree
- Somewhat agree
- Somewhat disagree
- Disagree

Statements:

- I would be afraid of being discriminated against if I offered an abortion.
- I would be afraid of being judged negatively by friends or family if I offered an abortion.
- I would be afraid of legal repercussions if I offered an abortion.
- I would be afraid of abortion opponents if I offered an abortion.
- I would be afraid of losing patients if I offered an abortion.
- Medical personnel should be required to refer patients to a trained professional if they do not want to perform an abortion themselves

**Page 15**

**Thank you for your Participation!**

We would like to thank you very much for your help.

Your answers have been saved; you can now close the browser window.
